# The Complex Relationship Between Sleep Quality and Job Satisfaction: A Machine Learning-Based Bayesian Rule Set Algorithm

**DOI:** 10.3390/bs15030276

**Published:** 2025-02-26

**Authors:** Xin Liu, Nan Qin, Xiaochong Wei

**Affiliations:** School of Public Administration and Policy, Renmin University of China, Beijing 100872, China; lxin@ruc.edu.cn (X.L.); qinnan@ruc.edu.cn (N.Q.)

**Keywords:** WNS framework, conservation of resources theory, job satisfaction, sleep quality, Bayesian rule set

## Abstract

In today’s highly competitive and rapidly evolving work environment, employee job satisfaction is a crucial indicator of organizational success and employee well-being. Utilizing the Bayesian rule set (BRS) algorithm, this study systematically explored how multiple variables, such as sleep quality, autonomy, and working hours, interact to influence job satisfaction. Based on an analysis of 618 data points from the CGSS database, we found that a single variable alone is insufficient to significantly improve job satisfaction: instead, a combination of multiple factors can substantially enhance it. Specifically, individuals who are older, have medium to high levels of sleep quality, and work fewer hours report higher job satisfaction. Similarly, individuals with medium to high health levels, high autonomy, and shorter working hours also exhibit high job satisfaction. By employing a multivariable combination analysis approach, this study reveals the complex pathways that affect job satisfaction, providing new theoretical insights and practical guidance for organizations seeking to improve employee satisfaction.

## 1. Introduction

In recent years, sleep has emerged as a critical factor influencing employee well-being and organizational outcomes. Existing studies have demonstrated that sleep significantly impacts not only individuals’ physical and mental health but also their work behavior and job satisfaction (Lin et al., 2018; Sheng et al., 2018). Sleep encompasses multiple dimensions, including sleep quality (perceived satisfaction with sleep), sleep duration (total time spent sleeping), sleep efficiency (proportion of time in bed spent sleeping), sleep continuity, and sleep variability (Barnes et al., 2023; Litwiller et al., 2017). Among these dimensions, both sleep quality and sleep duration have been consistently linked to workplace outcomes, with some research suggesting that sleep quality may have stronger associations with daytime functioning than sleep duration alone (Pilcher et al., 1997; Sonnentag et al., 2008).

However, most prior research has taken a single-variable approach, failing to capture the complex interplay between sleep and multiple organizational and personal factors. Studies suggest that work-related factors (e.g., autonomy, work hours, job stress) and nonwork-related factors (e.g., social support, family responsibilities) jointly shape sleep, which in turn influences employees’ job satisfaction (Crain et al., 2018; Van Laethem et al., 2015; Hobfoll, 2011). Job satisfaction—defined as an employee’s evaluative judgment about their job or job situation (Weiss, 2002)—differs from broader work attitudes such as organizational commitment or work engagement in its specific focus on the job itself rather than the organization or work in general (Judge & Kammeyer-Mueller, 2012). We focus specifically on job satisfaction rather than broader work attitudes because it has been most consistently linked to sleep in previous research (Litwiller et al., 2017) and represents a critical outcome for both individual well-being and organizational success (Judge et al., 2017).

The relationship between sleep and job satisfaction exhibits considerable complexity, characterized by potential bidirectional influences and contextual dependencies. While some research positions sleep as an antecedent of job satisfaction (Litwiller et al., 2017), other studies suggest that job satisfaction itself may shape sleep patterns, creating a reciprocal relationship (Qiu et al., 2022). This bidirectional influence complicates the identification of clear causal pathways. Our study did not attempt to definitively resolve this causal ambiguity, but rather aimed to identify complex patterns of variable combinations that reliably predict job satisfaction, which could inform future longitudinal research on causal mechanisms. Additionally, emotional labor further complicates this relationship, as it both affects and is affected by sleep (Yeh et al., 2020). Despite these findings, current research methods struggle to effectively identify the intricate pathways through which these factors interact to influence job satisfaction (Li, 2017).

The work, nonwork, and sleep (WNS) framework, proposed by Crain et al. (2018), provides a theoretical foundation for understanding how sleep mediates the relationship between work and nonwork domains. In this framework, sleep is conceptualized as an overarching construct encompassing multiple dimensions, including quality, duration, and timing. The WNS framework emphasizes that sleep serves as a crucial mechanism for managing energy and time resources, thereby influencing both workplace performance and personal well-being. However, the framework focuses primarily on broad domain interactions and lacks a detailed explanation of the specific mechanisms through which sleep influences resource allocation and job satisfaction. For instance, it remains unclear how variations in different dimensions of sleep translate into changes in workplace attitudes and behaviors, and empirical validation of these pathways is still insufficient (Crain et al., 2018).

Complementing the WNS framework, the conservation of resources (COR) theory offers a resource-based perspective on stress and well-being. This theory posits that individuals actively strive to acquire, protect, and retain resources to cope with stress (Hobfoll, 1989). According to Hobfoll (1989) and Hobfoll et al. (2018), resource depletion—such as long work hours, work–family conflict, or job insecurity—can reduce sleep quality and duration, leading to lower job satisfaction. Conversely, resource gains—such as autonomy, social support, or financial stability—can mitigate stress, improve sleep, and enhance job satisfaction. However, the existing research has not fully explored the role of sleep as a dynamic resource within the COR framework, leaving gaps in our understanding of how different sleep dimensions interact with resource accumulation and depletion in shaping job satisfaction.

Despite the increasing recognition of sleep as a key driver of organizational outcomes, systematic research on the complex pathways linking sleep and job satisfaction remains limited. This study sought to address this gap by examining how multiple factors—such as social status, autonomy, and work hours—interact with sleep dimensions (particularly sleep quality and sleep duration) to influence job satisfaction. To achieve this, we employed the Bayesian rule set (BRS) methodology, an advanced machine learning technique capable of uncovering complex nonlinear interactions among multiple variables. Unlike traditional regression models, which assume linear relationships, BRS allows for the identification of multivariable combinations that jointly predict job satisfaction, making it particularly suited for analyzing dynamic workplace factors without making strong assumptions about causality.

This study integrated the WNS framework and COR theory to identify the rule sets formed by the interactions between sleep and various work and nonwork factors that influence job satisfaction. The findings will contribute to a deeper theoretical understanding of the role of sleep in employee well-being and provide practical recommendations for organizations to improve job satisfaction through better management of sleep-related resources.

## 2. Literature Review and Analytical Framework

The relationship between sleep and job satisfaction has been examined from various perspectives, leading to complex and diverse findings. Most studies support a positive correlation between sleep quality and job satisfaction, indicating that employees with higher sleep quality generally report greater job satisfaction. However, the mechanisms underlying this relationship remain inconsistent. Some research suggests that sleep directly enhances job satisfaction. For example, Barnes et al. (2023) found that better sleep quality improves work behavior and productivity, which subsequently increases job satisfaction. Other studies propose that the effect of sleep on job satisfaction is indirect, operating through psychological mechanisms. Opoku et al. (2023) demonstrated that sleep enhances psychological capital while reducing burnout, which in turn leads to higher job satisfaction. Furthermore, some scholars argue that the effect of sleep on job satisfaction is context-dependent, with moderating variables influencing the strength of this relationship. For instance, Ye et al. (2023) found that in high-adaptability work environments, the positive impact of sleep on job satisfaction is amplified, highlighting the role of contextual factors.

Despite the general consensus that sleep positively impacts job satisfaction, several unresolved issues persist in the literature. These inconsistencies can be categorized into three key areas. First, the issue of causal ambiguity remains a major challenge. Some studies argue that sleep is an antecedent of job satisfaction (Litwiller et al., 2017), while others suggest that job satisfaction itself may shape sleep, creating a bidirectional relationship (Qiu et al., 2022). This reciprocal influence complicates the identification of clear causal mechanisms. Second, sample heterogeneity contributes to inconsistencies in findings. Differences in gender, age, health conditions, and job characteristics may lead to variations in how sleep affects job satisfaction (Benjamins et al., 2017). Without adequately accounting for these differences, results across studies may lack comparability. Third, the omission of key variables weakens the robustness of existing research. While many studies identify moderating or mediating factors, they often overlook crucial control variables. For example, Opoku et al. (2023) identified psychological capital and burnout as mediators, but did not account for work–family conflict or working hours, both of which are known to impact sleep and job satisfaction. Additionally, debates persist regarding the magnitude of these effects, with some studies reporting strong mediating or moderating relationships, while others find only weak or inconsistent effects (Henderson & Horan, 2021; Litwiller et al., 2017).

To address these challenges, this study integrated the conservation of resources (COR) theory and the work, nonwork, and sleep (WNS) framework. From the COR perspective, sleep represents a critical psychological and physiological resource, influencing employees’ ability to manage stress and maintain well-being. When individuals experience resource depletion, such as excessive work demands or work–family conflict, their sleep deteriorates, leading to lower job satisfaction (Hobfoll, 1989). Conversely, when employees gain or conserve resources, such as autonomy or social support, sleep improves, which enhances job satisfaction (Hobfoll, 1989; Hobfoll et al., 2018). The WNS framework further expands on this by emphasizing the interactive nature of sleep with work and nonwork domains. While it acknowledges that sleep precedes job satisfaction, it also highlights the importance of examining complex interactions between work demands, personal life responsibilities, and sleep-related factors.

To address sample heterogeneity, this study incorporated both work-related and nonwork-related factors into its analysis, exploring how different individual and contextual variables moderate or mediate the relationship between sleep and job satisfaction. Furthermore, to mitigate the issue of omitted variables, this study identified previously overlooked factors that may play a role in shaping the sleep–satisfaction relationship. By integrating both COR theory and the WNS framework, this study aimed to provide a more comprehensive and nuanced understanding of the mechanisms linking sleep and job satisfaction.

### 2.1. The WNS Framework and Conservation of Resources (COR) Theory

In this study, COR theory is integrated with the WNS framework to systematically analyze the complex interactions between work factors, nonwork factors, and sleep quality and their influence on job satisfaction. The WNS framework focuses on the interactions between work, nonwork, and sleep, while COR theory provides a perspective on the dynamic changes in resources that help explain these interactions. Specifically, key variables in this study, such as working hours, autonomy, and work–family conflict, affect an individual’s resource consumption and conservation, interacting with sleep quality to impact job satisfaction. The introduction of COR theory makes the selection and analysis of variables within the WNS framework more systematic and comprehensive, facilitating a more precise understanding of how the dynamic changes in resources within the work environment affect employees’ mental health and job performance.

#### 2.1.1. Overview of the WNS Framework

The work, nonwork, and sleep (WNS) framework, proposed by Crain et al. (2018), is a comprehensive theoretical model designed to integrate the relationships among work, nonwork, and sleep, which are three essential domains of life. The framework emphasizes that traditional work–nonwork interface research often neglects sleep, a critical factor that plays a vital role in both work and nonwork activities. Ignoring the effects of sleep quality prevents a full understanding of attitudes, behaviors, and states within the work and nonwork domains. The core concepts of the WNS framework include the following. (1) Sleep Quality and Quantity: Sleep encompasses not only duration (sleep quantity) but also sleep quality, such as difficulty falling asleep, maintaining sleep, and frequency of nighttime awakenings. High-quality and sufficient sleep is crucial for daily performance and health. (2) Resource Mechanisms: The WNS framework highlights human energy and time as key resource mechanisms. Adequate sleep restores an individual’s physical energy and vitality, providing more time resources for work and nonwork activities. (3) Interaction Relationships: There are complex interactions among sleep, work, and nonwork activities. Sleep affects the performance of work and nonwork activities, while work and nonwork activities in turn influence the quality and quantity of sleep. (4) Contextual Factors: Individual characteristics, work tasks, and social support play important moderating roles in these interactions.

#### 2.1.2. Overview of the Conservation of Resources (COR) Theory

The conservation of resources (COR) theory, proposed by Hobfoll (1989), centers on the idea that people strive to acquire, conserve, and protect resources they consider valuable. The loss of resources is seen as a primary source of stress, while the acquisition and conservation of resources help individuals cope with stress, enhancing their mental health and job satisfaction. Resources include material resources (e.g., money, high-value tools), conditional resources (e.g., job security), personal resources (e.g., self-esteem, self-efficacy), and energy resources (e.g., time, physical energy) (Hobfoll, 2011; Hobfoll et al., 2018). When individuals face resource threats or actual resource losses, their stress levels significantly increase, negatively affecting mental health and job performance. Conversely, when individuals effectively conserve resources or strategically acquire new resources to offset resource depletion, their stress levels are alleviated, thereby improving job satisfaction and overall well-being (Hobfoll, 1989, 2011; Hobfoll et al., 2018).

### 2.2. Variable Selection Based on the WNS Framework and COR Theory

By integrating the dynamic resource perspective of COR theory with the interaction mechanisms of the WNS framework, we can better understand the key variables that play significant roles within this framework. These variables interact with sleep quality through the conservation and depletion of resources, ultimately influencing job satisfaction. The Bayesian rule set (BRS) is particularly suitable for this analysis, as it can identify important factors that interact with the key variables to impact job satisfaction, while also controlling for non-core variables not directly relevant to the study.

It is important to note that the BRS model does not rely on traditional statistical assumptions, such as linear relationships or normally distributed errors. Instead, the model reveals complex nonlinear patterns in the data rather than providing parameter estimates of independent variable contributions, thereby avoiding strict assumptions.

#### 2.2.1. Work-Related Variables

Within the WNS framework, several key work-related variables shape the relationship between sleep quality and job satisfaction. These variables include working hours, autonomy, work–family conflict (WFC), family–work conflict (FWC), commute time, and income. Each of these variables interacts with sleep quality through distinct resource mechanisms, which collectively influence job satisfaction through their impact on resource depletion and conservation.

Working hours demonstrate a significant negative correlation with sleep duration, particularly in cases of extended work time. From the perspective of COR theory, extended work hours lead to rapid resource depletion, and the lack of sufficient recovery time exacerbates this depletion, reducing job satisfaction (Hobfoll et al., 2018). According to the WNS framework, long working hours not only compress sleep time but also increase fatigue and psychological stress, further affecting job satisfaction. Research has shown that increased work hours often lead to reduced sleep and heightened work-related stress and fatigue, which in turn diminish sleep quality and overall health, thereby affecting job satisfaction (Artazcoz et al., 2009; Hsu et al., 2019).

The level of control and independence employees have in their work significantly influences their resource management capabilities. In COR theory, high autonomy helps buffer resource loss and promotes resource conservation and acquisition, leading to a resource gain cycle that enhances job satisfaction (Hobfoll, 1989, 2001; Hobfoll et al., 2018). The WNS framework suggests that autonomy positively influences sleep quality and job satisfaction by reducing time pressure and increasing control over work schedules. Studies have shown that employees with higher autonomy can better manage their work and nonwork time, ensuring adequate sleep. This autonomy not only reduces stress but also improves job satisfaction (Boxall & Macky, 2014; Wheatley, 2017; Brossoit et al., 2020).

Both WFC and FWC negatively affect sleep quality and job satisfaction by competing for time and energy resources. The WNS framework indicates a significant positive correlation between WFC and sleep deprivation and insomnia symptoms. WFC increases work-related stress, which lowers job satisfaction (Jia & Fu, 2022) and also leads to poorer sleep quality (Buxton et al., 2016), causing anxiety and stress when dealing with conflicts, which in turn affects job satisfaction (Zhang et al., 2017). Similarly, family responsibilities’ influence on work performance through FWC reduces job satisfaction (Namasivayam & Mount, 2004), increasing psychological burdens and impacting sleep quality. COR theory further explains this phenomenon, suggesting that frequent resource depletion makes it difficult for individuals to maintain resource stability, leading to a vicious cycle of resource loss, poorer sleep quality, and lower job satisfaction (Hobfoll, 1989, 2014; Hobfoll et al., 2018).

Commuting duration significantly impacts resource allocation between work and rest. According to COR theory, long commutes lead to resource depletion, making it difficult to maintain a balance between work and rest (Hobfoll, 2014). This continuous resource consumption reduces job satisfaction, as individuals lack sufficient resources to recover and maintain both mental and physical balance while dealing with work-related stress. The WNS framework emphasizes that commute time directly impacts sleep and job satisfaction, as commuting competes with sleep time. Longer commutes increase fatigue and reduce rest time, leading to higher fatigue levels and lower job satisfaction (Han et al., 2022).

Income serves as a crucial resource that indirectly affects sleep quality through its influence on perceived social status and job satisfaction. According to COR theory, high income, an important resource, helps individuals more effectively maintain and acquire other resources, reducing the risk of resource depletion and enhancing overall well-being and job satisfaction (Hobfoll, 1989). High income is also associated with a higher perception of social status and job satisfaction, which improves sleep quality. According to the WNS framework, higher income alleviates financial stress, improves sleep quality, and consequently enhances job satisfaction (Wang et al., 2022).

#### 2.2.2. Nonwork-Related Variables

The WNS framework emphasizes three critical nonwork variables that significantly influence the sleep–satisfaction relationship: social class, perceived fairness, and trust in others. These variables function as important resources that can either facilitate or hinder an individual’s ability to maintain quality sleep and job satisfaction.

Social class affects individuals’ access to resources and their perception of stress, significantly influencing sleep quality and job satisfaction. From the perspective of COR theory, the elevation of social class often comes with increased opportunities to acquire resources, allowing individuals to better cope with resource depletion, thereby maintaining higher levels of mental health and job satisfaction (Hobfoll, 1989). Employees in higher social classes tend to enjoy better working conditions and development opportunities, as well as superior living conditions and sleep environments, all of which enhance job satisfaction (De Moortel et al., 2014; Framke et al., 2019; McGuffog et al., 2023).

Perceived fairness functions as a psychological resource that influences both sleep quality and job satisfaction. According to COR theory, fairness helps individuals stabilize their mental state, reducing resource loss and stress caused by perceived unfairness (Hobfoll, 2011). This resource management ability enables individuals to better cope with the risks of resource depletion, maintaining mental health and improving overall job satisfaction. The WNS framework also highlights the critical role of fairness in job satisfaction and mental health. Studies have shown that employees who perceive fairness experience lower stress and better sleep quality, which in turn enhances job satisfaction (Song et al., 2020).

Trust in others serves as a vital social resource that positively affects sleep quality and job satisfaction by increasing social support and reducing psychological stress. According to COR theory, social support acts as an external resource that provides additional assistance beyond the individual’s own resources. In situations involving work-related stress or the common pressure of balancing work and personal life, social support effectively buffers the exhaustion of resources, helping individuals better cope with life’s challenges (Hobfoll, 1989; Hobfoll et al., 2018; Hobfoll & Wells, 1998). Trust in colleagues and management enhances job satisfaction and team collaboration (Costigan et al., 2011). A trusting environment not only reduces work-related tension and anxiety but also improves sleep quality, thereby further increasing job satisfaction (Hsieh et al., 2021).

#### 2.2.3. Sleep-Related Variables

The WNS framework identifies two primary sleep-related variables that directly influence job satisfaction: sleep duration (quantity) and sleep quality. These variables represent distinct, yet interrelated aspects of sleep that serve as crucial resources for maintaining job satisfaction.

Sleep duration represents the quantitative aspect of sleep, directly affecting job satisfaction and overall health. According to the WNS framework, sleep quantity reflects the total time spent sleeping, while sleep quality captures the effectiveness of sleep. Sufficient and high-quality sleep not only restores physical and mental energy but also improves job performance and satisfaction. Good sleep quality helps reduce anxiety and depression levels, minimizes fatigue, and enhances job satisfaction (Sonnentag et al., 2008; Silva et al., 2022). COR theory also considers sleep quality an important restorative resource, helping individuals avoid rapid resource depletion. This in turn helps maintain high levels of resource reserves when facing work-related stress, contributing to better job performance and improved job satisfaction (Hobfoll et al., 2018).

### 2.3. Non-Core Variables

To effectively isolate the impact of sleep quality on job satisfaction, this study incorporated several demographic and contextual variables as non-core control variables. These variables, while not central to our theoretical framework, have been consistently shown in previous research to influence both sleep patterns and job satisfaction. Drawing from the existing literature (Shi & Long, 2018; Lin et al., 2018; Crain et al., 2018), we identified six key non-core variables: marital status, gender, part-time job status, age, education level, and health status.

Marital status and gender represent fundamental demographic factors that shape sleep and work experiences. Married individuals often experience different sleep and job satisfaction outcomes due to the complex interplay of family responsibilities and spousal support. Gender differences manifest in varying patterns of work-related stress and family responsibilities, potentially affecting both sleep quality and job satisfaction.

Employment characteristics, particularly part-time job status and age, introduce additional complexity to the sleep–satisfaction relationship. Part-time employment can create unique time management challenges that impact sleep quality, while age-related variations in sleep needs and career expectations influence job satisfaction levels across different life stages.

Socioeconomic factors, specifically education level and health status, serve as important control variables. Education level often correlates with perceived social status and career opportunities, thereby influencing job satisfaction. Health status maintains particularly strong associations with both sleep quality and job satisfaction, with poorer health typically corresponding to lower sleep quality and decreased job satisfaction.

These non-core variables have been well documented in previous research (Onuoha & Idemudia, 2018; Carrillo-García et al., 2013; Kim et al., 2008; Lee & Wilbur, 1985; Bae, 2021), and their inclusion helps isolate the unique effects of our primary variables of interest while controlling for potentially confounding influences.

### 2.4. Summary and Research Objectives

This study integrated the work, nonwork, and sleep (WNS) framework and conservation of resources (COR) theory to systematically examine how various factors interact with sleep quality to influence job satisfaction. While prior research has established a general link between sleep quality and job satisfaction, inconsistencies persist due to issues such as causal ambiguity, sample heterogeneity, and the omission of key control variables. To address these gaps, this study adopted a resource-based perspective to explore the mechanisms through which work-related and nonwork-related factors contribute to sleep quality and in turn affect job satisfaction.

To achieve this, the study focused on three key categories of variables. Work-related variables included working hours, autonomy, work–family conflict, family–work conflict, commute time, and income, with income serving as a crucial resource that influences both sleep quality and job satisfaction through its impact on perceived social status and stress levels. Nonwork-related variables, including social class, fairness, and trust in others, influence sleep quality by affecting an individual’s access to psychological and social resources. Sleep-related variables encompass two primary dimensions—sleep quality and sleep duration (quantity)—both of which play direct roles in determining job satisfaction and overall well-being. Beyond these, demographic factors such as income, age, gender, marital status, and health status were treated as non-core variables to control for potential confounding effects.

To uncover the complex nonlinear interactions among these variables, this study employed the Bayesian rule set (BRS) methodology, an advanced machine learning approach that identifies patterns of variable combinations rather than assuming linear relationships. Unlike traditional regression models, which may oversimplify dynamic workplace interactions, BRS enables a more nuanced analysis of how different factors collectively influence sleep quality and job satisfaction.

By bridging theoretical insights with advanced analytical techniques, this study aimed to contribute to a deeper understanding of the mechanisms underlying the sleep–job satisfaction relationship. The findings will not only enhance theoretical frameworks but also offer practical implications for organizations, providing data-driven strategies to improve employee well-being, optimize resource management, and enhance job satisfaction.

## 3. Research Design

### 3.1. Sample and Data

All data used in this study were derived from the 2021 resident questionnaire section of the Chinese General Social Survey (CGSS) database. The CGSS is China’s earliest nationwide, comprehensive, and continuous academic survey project, and is conducted by the Chinese Survey and Data Center at Renmin University of China. Following international standards, the CGSS has been conducted annually since 2003, surveying over 10,000 households across provinces, municipalities, and autonomous regions in mainland China each year. Each round of CGSS data is independent cross-sectional data, reflecting the social conditions at the time of the survey, rather than tracking the same respondents over time. Therefore, the design and implementation of CGSS are more suited for cross-sectional data analysis.

The original sample size of CGSS 2021 was 8148 respondents. To improve the quality of the data, variables with more than 10% missing data were excluded from the analysis. After removing outliers (for instance, cases where the reported work time was 24 h per day or more than 84 h per week), a total of 618 valid samples were retained. To further enhance the robustness of our analysis, we conducted an additional test by removing variables with more than 5% missing data, which yielded 497 valid samples. The results remained consistent across both sample sizes, demonstrating the robustness of our findings.

This study primarily investigated the impact of the combination of sleep quality and other variables on job satisfaction. The descriptive statistical results provide the basic characteristics of these variables, as shown in Table 1. The sleep quality scores range from 1 to 4, with a mean of 2.034 and a standard deviation of 0.629, indicating that most respondents rate their sleep quality as low. Job satisfaction scores range from 1 to 5, with a mean of 2.304 and a standard deviation of 0.816, suggesting that respondents generally have low satisfaction with their current jobs. For the categorical variables, the sample demographics show that 77.0% of respondents were married (n = 476) and 23.0% were single (n = 142). The gender distribution was nearly equal, with 49.8% female (n = 308) and 50.2% male (n = 310). Regarding education levels, 11.7% had below high school education (n = 72), 39.3% had high school or equivalent education (n = 243), 5.5% held associate degrees (n = 34), 13.4% held bachelor’s degrees (n = 83), and 30.1% had graduate degrees or above (n = 186). In terms of employment status, the vast majority of respondents (92.9%, n = 574) were employed full time, while 7.1% (n = 44) held part-time positions. The variables used in this study and their corresponding original survey items are presented in Table 1.

### 3.2. Research Methodology

Traditional analytical methods, such as regression analysis and qualitative comparative analysis (QCA), have certain limitations in handling multivariable combinations and complex nonlinear relationships. Regression analysis assumes linear relationships between variables, which may introduce bias when dealing with real-world complex interactions. While QCA is capable of addressing multivariable combinations, its performance and computational efficiency may be compromised when dealing with large datasets and high heterogeneity. Chiu and Xu (2023) indicated that QCA performs well with small-sample data, but with large datasets, it may face challenges in interpretability and computational feasibility. To overcome these limitations, this study adopted the Bayesian rule set (BRS) method proposed by Chiu and Xu (2023). BRS is an emerging machine learning algorithm with the following advantages.

1. Handling large-scale and noisy data: BRS can manage complex data with substantial noise and heterogeneity without discarding crucial information. Unlike QCA, BRS leverages the variability in the data without excluding observations due to random errors.
2. Avoiding overfitting: By balancing in-sample fit and model complexity directly, BRS prevents overfitting and enhances the model’s generalizability. The BRS algorithm can identify a more concise and efficient set of rules while ensuring interpretability.
3. Improved interpretability: BRS classifies observations using rule sets, where the rules consist of conditions linked by logical operators (e.g., if Condition A and Condition B are true, or if Condition C is true, then Y is true). This approach enhances interpretability by clearly revealing the complex interactions between variables.
4. Nonlinear relationships: BRS uncovers complex nonlinear relationships within the data, making it more suitable than traditional regression analysis for handling intricate interaction effects found in real-world data. By employing the BRS method, higher-order interactions and other complex relationships between variables can be better understood and explained.
5. Computational efficiency: Even with large samples and highly heterogeneous data, BRS provides an efficient and interpretable solution. Compared to QCA, BRS does not lose information when dealing with random errors in the data and balances complexity and performance to find a more concise and efficient solution.

Additionally, this study compared BRS with other common machine learning methods, such as LASSO, decision trees, and random forests.

### 3.3. Data Processing

Survey data are particularly suitable for Bayesian rule set (BRS) analysis because survey responses are often ordinal or binary. Following the method proposed by Chiu and Xu (2023), binary features were created for the variables by breaking down ordinal variables into fewer, overlapping incremental categories (e.g., “low”, “low or medium”, “high”). The maximum rule length was set to L=3 to prevent the candidate rule pool from growing exponentially as the number of variables increased, avoiding rules that are too long and difficult to interpret. This is the optimal choice. Additionally, limiting the rule length helps ensure the model’s interpretability and prevents overly complex rules that could lead to overfitting. The minimum support threshold was set to 5% (i.e., a rule must appear in at least 5% of the data). This threshold ensures that only rules with sufficient frequency and statistical significance are selected, reducing the influence of random or noisy data. The maximum number of rules was set to 5000. The study ran 10 chains with 10,000 iterations per chain to ensure the model’s stability and convergence. Running multiple chains and a large number of iterations helps avoid the model getting stuck in local optima, ensuring that the global optimum is found.

To ensure the applicability and consistency of the data, the original dataset underwent systematic preprocessing. First, to align the scoring direction, the study reversed the scoring for three variables: autonomy, sleep quality, and job satisfaction. This treatment ensures that higher scores represent higher levels of the variables. To extract columns with only two unique values, a logical filtering method was used to create a new data frame, X, which contains only the binary variables from the original dataset. Next, job satisfaction was discretized by setting values greater than the mean plus one standard deviation as the high-job-satisfaction group, while other values were assigned to the low-job-satisfaction group. For instance, the age variable was discretized according to the 1/3 and 2/3 quantiles, resulting in four categories: low, low-–medium, medium–high, and high. Health status was divided into four categories based on ratings: low, low–medium, medium–high, and high. Income was also discretized using the 1/3 and 2/3 quantiles, resulting in four categories: low, low–medium, medium–high, and high. Other variables, such as social class, trust in others, fairness, autonomy, sleep quality, weekday sleep time, commute time, work–family conflict, and family–work conflict, were similarly discretized and categorized.

## 4. Results Analysis

### 4.1. Descriptive Statistical Analysis

This study primarily investigated the impact of the combination of sleep quality and other variables on job satisfaction. The descriptive statistical results provide the basic characteristics of these variables, as shown in Table 2. The sleep quality scores range from 1 to 4, with a mean of 2.034 and a standard deviation of 0.629, indicating that most respondents rate their sleep quality as low. Job satisfaction scores range from 1 to 5, with a mean of 2.304 and a standard deviation of 0.816, suggesting that respondents generally have low satisfaction with their current jobs. Additional details about other variables can be found in Table 2.

### 4.2. Prevalence and Coverage Analysis

**Figure 1:**
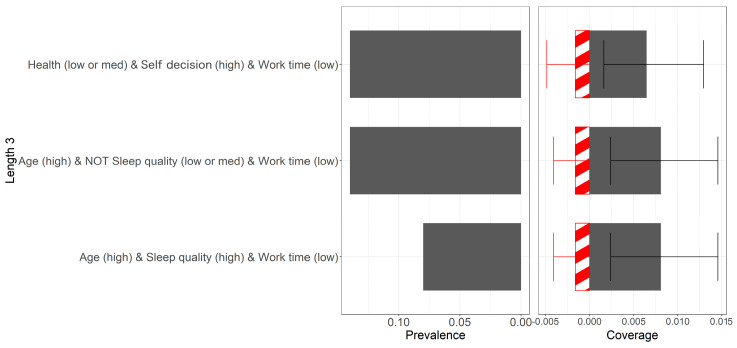
Prevalence and coverage of rules (note: for abbreviations used in the figure, please refer to Table 1 for corresponding full variable names).

Figure 1 displays the prevalence and coverage of each rule in a bar chart, allowing a visual understanding of how frequently each rule appears and its coverage proportion across different samples. On the left side of Figure 1, the prevalence of rules is shown. Prevalence refers to the proportion of times a rule appears across different samples (for instance, in bootstrapping, the frequency with which a particular rule occurs). A rule is considered to have high prevalence if it appears in at least 5% of the samples. For example, if a rule appears in at least 5 out of 100 bootstrap samples, it qualifies as a high-prevalence rule. Conversely, if a rule appears in less than 5% of the samples, it is classified as low prevalence. This means that if a rule appears fewer than 5 times in 100 bootstrap samples, it has low prevalence. Rules with high prevalence indicate that they are frequently selected across different sample draws, suggesting that the rule is stable and reliable overall. By analyzing the bar chart in Figure 1, high-prevalence and low-prevalence rules can be clearly identified.

On the right side of Figure 1, the coverage of rules is displayed, representing the number of samples that a rule (or rule set) can correctly classify. Rules with high coverage show consistent performance across different samples, usually accompanied by narrower error bars, indicating that the rule covers a larger portion of the data sample. In contrast, rules with low coverage classify fewer data points and are associated with wider error bars, suggesting that the performance of these rules varies significantly across different samples. Coverage is divided into true positives (correct classifications) and false positives (incorrect classifications). In the bar chart, positive values represent true positives, while negative values represent false positives (hence the appearance of negative coverage). The level of coverage is compared based on the bar chart in Figure 1. In the prevalence and coverage analysis, the rules highlighted in darker colors are those used in the final aggregated rule set (all rules shown in Figure 1 are in darker colors). By analyzing the bar chart on the right of Figure 1, one can evaluate the consistency and stability of each rule’s coverage across different samples.

The error bars on the right of Figure 1 represent the confidence interval (CI) of the coverage, indicating the variability in coverage across different samples. The upper and lower bounds of the error bars represent the 2.5th and 97.5th percentiles of coverage, meaning there is a 95% confidence level that the coverage falls within this range. The width of the error bars reflects the uncertainty in coverage: the wider the error bars, the greater the variability in coverage across samples, suggesting lower stability for the rule. Conversely, narrower error bars indicate more consistent performance across samples, implying higher stability. Bar charts with error bars showcasing coverage help evaluate the stability and reliability of the rules. High prevalence and narrow error bars in coverage suggest that a rule remains consistently important across different samples, making it a reliable predictor. Since negative coverage appears in Figure 1, negative error bars are also present. By examining the bar chart on the right of Figure 1, one can assess the consistency and stability of each rule’s coverage across different samples. This helps identify rules that are stable and effective in various contexts.

In Figure 1, it is evident that no single variable can predict high job satisfaction. Instead, a combination of multiple variables is required. For example, rule 1 consists of medium or high health status (health (med or high)), high autonomy (autonomy (high)), and low work time (work time (low)). This rule appears frequently in the sample, indicating that it is a common combination. In terms of coverage, this rule applies to a substantial portion of the sample, although the error bars are wide, suggesting relatively stable performance across samples. Rule 2 consists of older age (age (high)), sleep quality not lower than medium (not sleep quality (low or med)), and low work time (work time (low)). Rule 2 has the highest occurrence frequency and coverage in the sample, making it the most common combination. Notably, rule set 2 overlaps with rule set 3, which consists of older age (age (high)), high sleep quality (sleep quality (high)), and low work time (work time (low)). Thus, rule set 2 includes not only high sleep quality but also medium to high sleep quality. In the discussion, the focus is primarily on rule 1, as it is representative across various combinations.

### 4.3. Interaction and Rule Set Coverage Analysis

**Figure 2:**
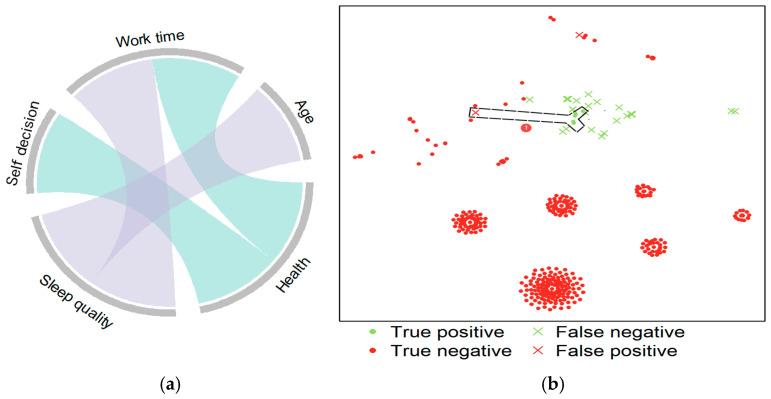
Interaction and rule set coverage. (**a**) Chord diagram representing variable interactions; (**b**) t-SNE plot showing data points covered by rule sets.

Figure 2a is a chord diagram used to illustrate the interactions between variables. The connections between nodes and chords visually represent how variables interact and form rule sets, showing how these interactions influence outcomes. In Figure 2a, each arc represents a variable. In this study, the variables may include age, autonomy, health (health status), work time, and sleep quality. The chords connecting the arcs indicate the interaction between variables: the more chords between variables, the more frequent their interaction. Variables connected by chords appear together in rule sets as joint conditions. The higher the number of chords between variables, the stronger their interaction. The colors in Figure 2a are purely for visual distinction and do not convey specific data meanings. The different colors of the arcs and chords help differentiate between various variables and interactions.

Figure 2a shows that individuals with medium or high health levels, high autonomy, and fewer work hours have higher job satisfaction (health (med or high) and autonomy (high) and work time (low)). Additionally, older individuals with medium to high sleep quality and fewer work hours also report higher job satisfaction (age (high) and NOT sleep quality (low or med) or work time (low)). Figure 2a further supports the conclusion from Figure 1 that rule set 2 overlaps with rule set 3. The results also show that work time plays a crucial role in both interaction patterns, highlighting the importance of reducing work hours to improve job satisfaction.

Figure 2b is a t-SNE plot, a technique used for visualizing high-dimensional data, primarily in exploratory data analysis. It displays the data points covered by the rule sets, helping to identify clusters and structures within the data and assess the effectiveness of the rules and the classification model’s accuracy. t-SNE reduces high-dimensional data into a two-dimensional space, making it easier to visualize the similarities between data points in high-dimensional space. After t-SNE dimensionality reduction, similar data points cluster together in the low-dimensional space. In Figure 2b, the clustering of data points covered by different rules can be observed. Each point in the t-SNE plot represents a data sample. Different colors and symbols in the t-SNE plot indicate different classification outcomes. True positives are represented by green circles, indicating data points correctly classified as positive. True negatives are shown as red circles, representing correctly classified negative samples. False negatives are represented by green X’s, indicating positive samples incorrectly classified as negative. False positives are shown as red X’s, representing negative samples incorrectly classified as positive.

By combining Figure 1 and Figure 2a, it is evident that the two rules, (health (med or high) and autonomy (high) and work time (low)) and (age (high) and NOT sleep quality (low or med) or work time (low)), are the most important. Both have relatively high prevalence, but (age (high) and NOT sleep quality (low or med) or work time (low)) has higher coverage. Additionally, both rules exhibit false positives, as shown by negative coverage in Figure 1. Further analysis of the t-SNE plot in Figure 2b reveals that the dashed box labeled “①” represents the rule (age (high) and NOT sleep quality (low or med) or work time (low)) well. The t-SNE plot demonstrates the distribution of data points covered by specific rules, validating the effectiveness of these highly prevalent and high-coverage rules across different data subsets. The data points encircled by the dashed lines represent samples that satisfy specific rules. The distribution and clustering of these points further confirm the high prevalence and coverage of these rules and also indicate the effectiveness of the model.

### 4.4. Robustness Test

**Figure 3:**
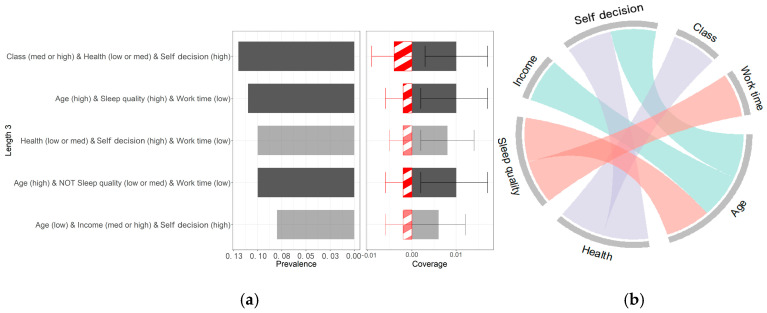
Prevalence, coverage, and interaction of rules. (**a**) Prevalence and coverage of rules; (**b**) rule interactions.

To enhance the robustness of the data validation in the algorithm, a deletion strategy was applied to variables with more than 5% missing data, resulting in a final dataset of 497 valid samples. After stricter sample screening, the program was rerun using the same parameter settings, and the results are shown in Figure 3.

In Figure 3, the darker-colored rule sets represent stronger importance. Three significant rule sets are clearly identified: ① health (med or high) and autonomy (high) and work time (low), ② age (high) and NOT sleep quality (low or med) or work time (low), ③ age (high) and sleep quality (high) and work time (low). Figure 3a,b show that age (high) and NOT sleep quality (low or med) or work time (low) and age (high) and sleep quality (high) and work time (low) continue to appear together, with rule set 2 still encompassing rule set 3. Figure 3b also reveals that age appears most frequently, followed by sleep quality, health status, and autonomy, indicating that these factors have a significant impact on job satisfaction. It should be noted that while rule set 1 has a higher prevalence, it also shows larger error margins in its coverage. Therefore, under stricter sample conditions, the conclusions of this study remain valid.

### 4.5. Comparison with Other Methods

The study followed the approach of Chiu and Xu (2023), comparing the Bayesian rule set (BRS) with LASSO, decision trees, and random forests. It is important to note that this comparison aimed to highlight the unique advantages of BRS in terms of model interpretability and applicability, rather than criticizing other methods.

#### 4.5.1. Comparison with LASSO

To encompass all model classes that BRS can describe (using rules of maximum length 3), a sixth-order interaction would be required. This would generate a matrix with billions of entries, exceeding the theoretical storage limits of software like RStudio (v.3.6.0). Therefore, the study limited variable selection to up to third-order interactions. The study generated interactions up to the third order and applied LASSO (using cross-validation to select the penalty parameter). Since this approach was exploratory, it resulted in a large number of non-zero terms, making interpretation difficult. The study then applied ordinary least squares (OLS) to the non-zero terms selected by LASSO, where 11 terms were found to be significant at the 5% level:
1. Age_lm_ × Famw_1_ × ${Workf}_{h}(<0)$, 2. Age_mh_ × Fair_h_ × ${Sleepq}_{h}(>0)$,3. Class_1_ × Health_h_ × Selfd_h_(<0),4. Class_mh_ × Income_1_ × ${Workf}_{l}(>0)$,5. Edu_h_ × Income_1_ × ${Roadt}_{h}(<0)$,6. Fair_1_ × Health_mh_ × ${Income}_{lm}(<0)$,7. Fair_1_ × Income_1_ × ${Sleepq}_{h}(<0)$,8. Famw_mh_ × Income_1_ × ${Selfd}_{l}(<0)$,9. Famw_mh_ × ${Roadt}_{h}\times {Sleept}_{h}(<0)$,10. Gender_1_ × Health_1_ × ${Roadt}_{lm}(<0)$,11. ${Selfd}_{l}$ × Trusto_lm_ × ${Workt}_{l}(<0)$



It is clear that the data description generated by regression differs from that of BRS. While it is theoretically possible to construct a linear model that has a similar meaning to the rule set, regression methods often fail to recover that model. Moreover, the descriptions provided by regression are generally less interpretable, as the substantive meaning of numerous significant interaction terms remains unclear. This observation alone demonstrates the practicality of rule sets (Chiu & Xu, 2023).

In addition to regression analysis, other methods can be used to understand high job satisfaction. This paper discusses the results obtained using decision trees and random forests. These methods were selected because decision trees are essentially another way of constructing rule sets, while random forests, an ensemble version of decision trees, are widely used in research of this kind (Chiu & Xu, 2023).

#### 4.5.2. Comparison with Decision Trees

**Figure 4:**
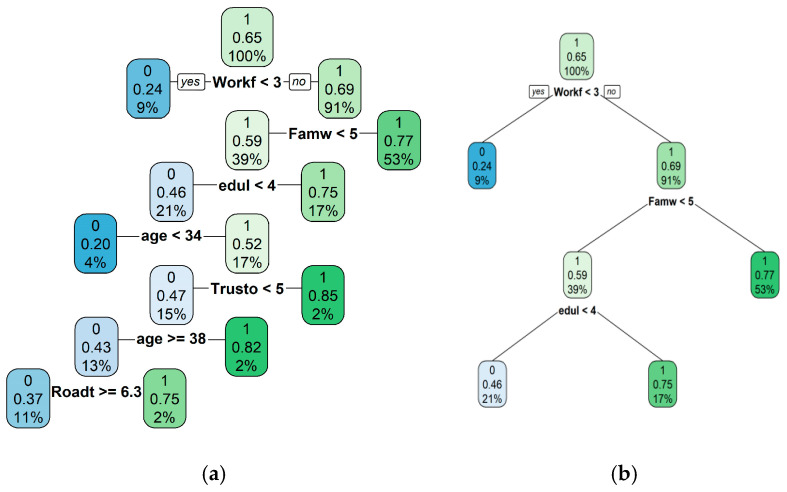
Decision tree analysis. (**a**) Complexity chosen via cross-validation; (**b**) similar complexity to aggregated BRS solution.

Each branch of a decision tree that ends with “1” represents a rule, and the union of these rules forms a rule set. For example, the tree in Figure 4b can be interpreted as: “work–family conflict ≥ 3 and family–work conflict ≥ 5, or work–family conflict < 5 and education level ≥ 4.” The complexity of this tree was carefully chosen to ensure that the resulting rule set was comparable to the aggregated BRS solution in terms of the number of rules and their average length.

Like BRS, this decision tree shares similar interpretations: low work–family conflict and low family–work conflict are considered important factors for predicting job satisfaction. However, the rule sets generated by decision trees are often less concise, and at the same level of simplicity, they tend to underperform compared to BRS (Chiu & Xu, 2023).

As shown in Figure 4a, when complexity is chosen via cross-validation, the resulting model is overly complex and less interpretable, potentially leading to overfitting. If complexity is chosen while maintaining model interpretability, a model similar to the one in Figure 4b may be obtained. However, this often produces a rule set that is worse than BRS because tree algorithms tend to be greedy, making locally optimal splits at each node, but rarely finding the global optimal solution (Chiu & Xu, 2023).

**Figure 5:**
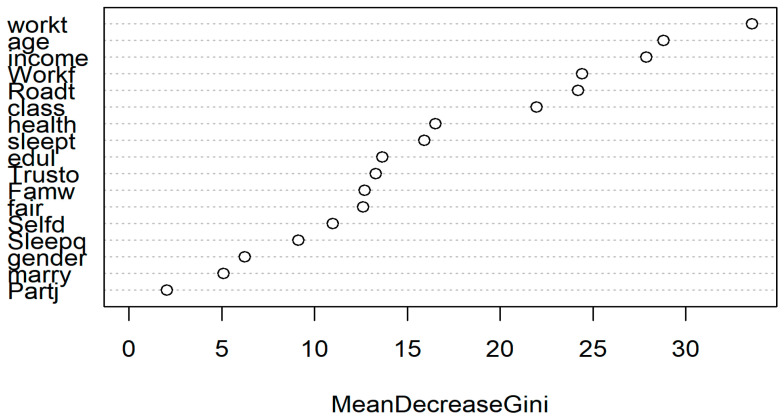
Assessing variable importance via random forest.

To address the overfitting issue, random forests can be considered. This method uses bootstrap aggregation (or “bagging”) to combine the predictions of multiple decision trees, each generated from different bootstrap samples, to form a final prediction. While this method has advantages in predictive performance, bagging sacrifices interpretability, because it is unclear which features of an observation led to its classification result. There are measures for assessing variable importance, but these do not reveal the interactions between variables. Figure 5 shows one measure of variable importance using random forests. It is possible to manually investigate variable interactions, but this approach can be redundant (since decision trees implicitly interact variables), which leaves it facing the same challenges as regression analysis (Chiu & Xu, 2023).

#### 4.5.3. Methodological Extension

In complex causal analysis, we typically explore how independent variables or configurations influence the dependent variable through a series of mechanisms. This section extends the complex mediation model proposed by Du et al. (2024), focusing on large-scale observational data (such as non-experimental or quasi-natural experiments), where the core independent variables coexist with numerous confounding factors. It introduces a method combining Propensity Score Matching (PSM) and Bayesian Rule Sets (BRS). This approach aims to address the limitations of QCA in large data with high noise and provides a more systematic approach to handling potential confounding variables.

The complex mediation model proposed by Du et al. (2024) combines configuration analysis with regression to identify the complex causal pathways of X → M → Y in multiple concurrent causal relationships. However, when dealing with large-scale observational data, particularly when the core independent variables are accompanied by many control variables, the current approach provides limited guidance on how to adjust and interpret these control factors. The key issue to address in this section is how to ensure proper adjustment and exclusion of potential confounders in large datasets.

Bayesian Rule Sets (BRS) provide an effective solution to these issues. BRS uses Bayesian inference to estimate the relationships between rules, enabling efficient processing of large datasets with more precise computations. By calculating probabilistic rules, BRS reveals the effects of condition combinations and avoids the computational complexity issues encountered in QCA when dealing with numerous combinations. Additionally, BRS can quantify the reliability and uncertainty of rules, offering more information and confidence for causal inference compared to the deterministic approach of QCA, thereby providing more flexible and robust causal inferences.

Furthermore, BRS excels at handling nonlinear relationships and complex interactions. Through Bayesian networks, BRS captures more complex causal paths between variables and considers interaction effects. This approach better models the impact of condition combinations on the outcome variable, whereas QCA typically only considers the sufficiency and necessity of conditions, making it less adept at handling complex interactions.

To address these issues, this section proposes a combined approach of PSM and BRS to match samples before configuration analysis, thus eliminating the influence of potential confounders. By applying PSM for propensity score matching, this approach ensures that the background variables of independent and control variables remain consistent across groups, improving the reliability and accuracy of the BRS analysis. Subsequently, the configuration analysis results from BRS will serve as a foundation to explore how independent variables affect the dependent variable through mediators in the complex mediation model framework.

Therefore, this section focuses on three key questions: First, how to effectively handle control variables and potential confounders in large-scale observational data to ensure the accuracy of regression analysis; second, whether matching (such as propensity score matching) should be performed before configuration analysis, or if the raw sample can be used directly for configuration; and third, how to maintain the interpretability and robustness of the model by incorporating complex mediation model thinking based on BRS output configurations.

##### Matching vs. Non-Matching: Two Approaches for Grouping Based on the Core Independent Variable

In non-experimental or quasi-experimental conditions, researchers can divide the sample into a “treatment group vs. control group” based on the core independent variable X (e.g., those above the mean plus one standard deviation are classified as the high group, and those below are classified as the low group) to simulate whether the sample is at a higher level of the independent variable. A large number of control variables W may influence the outcome Y or be related to X, so researchers can choose between two strategies: matching before configuration or directly using the raw sample for configuration (matching before discretization). The following sections describe these two methods.

First, following the concept of propensity score matching (PSM), the treatment probability in Equation (1) is constructed as:

(1) $$p_{i}=P\left(T_{i}=1|W_{i}\right)={logit}^{-1}\left(\beta_{0}+\beta_{1}W_{i1}+\cdots +\beta_{k}W_{ik}\right)$$

Then, within the treatment and control groups, samples with similar propensity scores $\beta_{i}$ are selected using nearest neighbor, radius, or caliper algorithms to obtain a matched subset. At this point, the treatment and control groups are more balanced with respect to the control variables, allowing subsequent configuration analysis to focus more on the mechanisms caused by the “high vs. low” differences in the independent variable X. The advantage of this approach is that it enhances causal inference and reduces bias from confounding factors. However, the downside is that a large number of samples may be discarded, and if the original sample is highly heterogeneous, the coverage of the subset may be compromised, limiting the depth of configuration analysis by BRS.

Next, we have inverse probability weighting (IPW). If researchers do not wish to lose samples or wish to maximize sample utilization, they can opt to use IPW. This method balances the control variables by weighting the samples rather than losing them through propensity matching. The basic idea of IPW is to calculate the propensity score for each observation and then weight the samples based on these probabilities. The specific weighting formula is shown in Equation (2):

(2) $${\mathrm{Weight}}_{i}=\frac{1}{p_{i}}\;\mathrm{if}\;T_{i}=1,\;\mathrm{or}\;\frac{1}{1-p_{i}}\;\mathrm{if}\;T_{i}=0$$

The advantage of this method is that it avoids losing samples and retains all observation data. However, if there is a large difference in control variables and significant noise, it may lead to extreme weights, which could affect the stability of the model and the reliability of the results. Therefore, when using IPW, researchers need to check the distribution of the weights to avoid extreme values.

Finally, there is configuration analysis without grouping. If researchers prefer not to divide the sample into treatment and control groups, they can directly use all observation data for configuration analysis without grouping. In this case, some control variables can be entered into the configuration tool along with the independent variable X (if they have significant interactions with X) or the control variables can be reserved for inclusion in the regression phase. The advantage of this approach is that it retains more information, and BRSs can search for a broader range of interaction combinations, thereby helping to capture more potential causal relationships.

##### Bayesian Rule Sets as a Replacement for QCA: Suitable for Large-Scale Observational Data

The Bayesian rule sets (BRSs) proposed by Chiu and Xu (2023) are based on Bayesian posterior:

(3) p(A∣S)∝π(A)×p(S∣A)

In Equation (3), π(A) uses a Poisson prior or similar methods to penalize the number and length of rules, allowing for a relatively concise “rule set” (configuration) to be identified in large samples with high noise. Compared to QCA, BRS does not require strict consistency thresholds and does not discard large numbers of cases when encountering contradictory configurations, offering better computational efficiency and robustness. For studies aiming to uncover multifactor concurrent causal relationships, BRS outputs logic forms such as “(Condition 1) AND (Condition 2) OR (Condition 3) THEN Y = 1.”

BRS, through the combination of priors and likelihoods, ultimately provides several rules $\left\{a_{1},a_{2},\ldots ,a_{M}\right\}$ for the positive class (e.g., high Y). Each rule $a_{m}$ takes the Boolean form: satisfying all the conditions in this rule (AND logic) means the rule is satisfied. Typically, BRS uses an OR combination, where if any one rule is satisfied, it predicts Y=1. For example, if there are two rules: ① Rule 1: (age ≥30) AND (work hours ≤40); ② Rule 2: salary ≥5000), then in BRS classification, satisfying either of these rules is considered a prediction of Y=1.

To map these rules to regression data columns, the most straightforward method is to construct an indicator variable $I_{m}$ for each rule $a_{m}$, which records whether each observation satisfies the rule. The expression is given by Equation (4):

(4) $$I_{m}(n)=\left\{\begin{array}{ll} 1, & \mathrm{if}\;\mathrm{observation}\;n\;\mathrm{satisfies}\;\mathrm{all}\;\mathrm{the}\;\mathrm{conditions}\;\mathrm{of}\;\mathrm{rule}\;a_{m}\\ 0, & \mathrm{otherwise} \end{array}\right.$$

For example, if Rule 1 is (Age ≥30) AND (Work Hours ≤40), for observation n, you check whether Age is ≥30 and Work Hours is ≤40. If both conditions are met, then $I_{1}(n)=1$, otherwise 0. This process is repeated for all observations (from 1 to N), resulting in a column $\left\{I_{1}(1),\ldots ,I_{1}(N)\right\}$, which is referred to as “Rule1_Indicator”. Similarly, for rules 2 through M, “Rule2_Indicator”, “Rule3_Indicator”, …, “RuleM_Indicator” are generated. These columns are then used as explanatory variables in the regression.

For example, if Rule 1 is defined as Age ≥30 AND Work Hours ≤40, for the n-th observation sample, we check whether Age is greater than or equal to 30 and whether Work Hours is less than or equal to 40. If both conditions are satisfied, the sample meets Rule 1, and we denote it as $I_{1}(n)=1$; otherwise, we denote it as $I_{1}(n)=0$. This check is repeated for all samples 1≤n≤N, resulting in a column vector containing the indicator variable for Rule 1, denoted as Rule1_Indicator. Similar checks are performed for other rules (e.g., Rule 2 to Rule M), generating Rule2_Indicator, Rule3_Indicator, and so on. These rule indicator variables will serve as independent variables in the regression analysis.

After completing the mapping process, the researcher can directly include the indicator variables for each rule along with the remaining control variables Z in a single-equation regression analysis. Of course, in the complex mediation model analysis, we face two scenarios: one with grouping and one without grouping.When propensity score matching (PSM) is applied to group the samples, the samples are typically divided into treatment and control groups. In this case, the grouping variable (i.e., treatment variable) will be included as an independent variable in the regression model. Suppose we use $I_{m}$ to represent the grouping variable, where $I_{m}=1$ indicates the treatment group (e.g., the high group) and $I_{m}=0$ indicates the control group (e.g., the low group). In this case, the regression model will consider the impact of $X_{m}$ on the mediator variable $X_{m}$ and further analyze the effects of M and $X_{m}$ on the outcome variable Y.

First, consider the impact of the rule set ${Rule}_{r}$ on the mediator variable M. The regression equation is shown in Formula (5):

(5) $$M=\alpha_{1}+\beta_{1}I_{m}+\gamma_{1}^{T}Z_{1}+\sum_{r=1}^{R}\lambda_{r}\;{\mathrm{Rule}}_{r}+u_{1}$$

where $I_{m}$ represents the treatment and control groups obtained through PSM. Z is used to exclude potential confounding factors. Rule**_r_** is the rule indicator variable generated by BRS. $u_{i}$ represents the unexplained portion in the model. Next, consider the regression equation for M on Y. Formula (6) expresses the effect of M on Y:

(6) $$Y=\alpha_{2}+\beta_{2}I_{m}+\lambda_{1}M+\gamma_{2}^{T}Z_{2}+u_{2}$$

Finally, we consider the joint effects of the rule set ${Rule}_{r}$ and M on the outcome variable Y. The regression equation in Formula (7) is as follows:

(7) $$Y=\alpha_{3}+\beta_{3}I_{m}+\lambda_{2}M+\gamma_{3}^{T}Z_{3}+\sum_{r=1}^{R}\theta_{r}\;{\mathrm{Rule}}_{r}+u_{3}$$

In the case where PSM grouping is not applied, we directly perform the rule set analysis. In this case, the term $\beta_{m}I_{m}$ is no longer needed, so it is omitted. By estimating the regression coefficients of the three paths, we can identify whether each rule has a significant impact on Y or M, and distinguish between direct and indirect effects (Baron & Kenny, 1986). The advantage of the optimized method is that by directly including control variables in the rule set analysis, we can more precisely estimate the direct and indirect effects of different rule sets, while effectively handling potential confounding factors. Additionally, by combining Propensity Score Matching (PSM) and Bayesian Rule Sets (BRS), this method not only improves the computational efficiency and robustness of the model but also quantifies the uncertainty of rules through Bayesian inference, providing more flexible causal reasoning and thus enhancing the interpretability and reliability of the model.

##### Improving the Approach to Complex Configurational Mediation: Greater Flexibility for Large-Scale Observational Data

If researchers prefer causal explanations and are concerned about substantial confounding factors, they can choose to “match before configuration”, ensuring that the treatment and control groups are more balanced with respect to control variables, thus allowing the BRS phase to focus more on the “high vs. low” differences in the core independent variable X. If preserving all samples and diversity is prioritized, matching can be skipped. In this case, BRS may uncover more complex rules, but stricter constraints on parameters such as rule length and support are needed, and control variables should be accounted for in the regression stage to compensate. While QCA is easier to implement in smaller datasets with fewer confounders, in large-scale datasets, consistency thresholds, conflicting configurations, and formulaic searches often lead to computational bottlenecks and increased model complexity. BRS, through prior regularization, can automatically prevent rule set overexpansion and does not require manually set thresholds to handle conflicting configurations, making it more suitable for diverse and high-noise data (Chiu & Xu, 2023). Although the proposed combination mining tool replaces QCA with BRS, it still incorporates mediators and remaining control variables in the final regression, distinguishing the direct and indirect effects of configurations on outcomes and mediators, in alignment with the original framework. If resources allow, both “raw sample + BRS + regression” and “matched subset + BRS + regression” can be executed in parallel to compare configuration solutions and results, evaluating the impact of matching on conclusions.

Through propensity score matching + BRS + complex mediation regression (optional), this section further extends the applicability of Du et al. (2024) in large-scale observational data. If researchers are more focused on causal inference and can accept sample exclusion, they can perform propensity matching to balance confounders. If exploration is more emphasized, matching can be omitted, but strict parameter settings for rule length, minimum support, and other factors should be enforced in the BRS phase.

##### Considerations and Optimization Strategies

First, researchers should pay close attention to the potential differences between the original BRS decision logic and the regression model. BRS typically uses an OR logic, where “if any rule is satisfied, predict the positive class.” However, in the regression equation, each rule corresponds to a coefficient for an independent variable, which is linearly summed. This approach can lead to a situation where if a sample satisfies multiple rules, the impact of these rules on the outcome is “counted multiple times” in the regression, deviating from BRS’s original decision logic of “predicting the positive class if any rule is satisfied”. Therefore, when interpreting the coefficients in the linear regression, researchers should be cautious about the conclusion regarding “the independent effect of a rule”, especially when there is significant overlap between rules or when the combinations of rules themselves have nonlinear relationships.

Secondly, when two or more rules are highly overlapping in the sample, multicollinearity may arise in the regression model. In such cases, the coefficients of the independent variables may show instability and variance inflation, making it difficult to accurately determine the contribution of a specific rule to the outcome. To mitigate this issue, researchers can combine or discard rules that are highly similar or have overly overlapping coverage, or retain only the most differentiated and representative core rules. This not only helps reduce the interference caused by multicollinearity in model estimation but also contributes to maintaining the interpretability of the model.

Additionally, the number of rules itself is a critical control point. Although BRS typically generates fewer rules than QCA, if researchers do not perform any selection, a large number of rules (e.g., ten or more) may still emerge. Including all rules indiscriminately in the regression model can lead to problems such as overly large dimensions and very low explanatory power, making it difficult for researchers to grasp the complex interactions at the interpretive level. To address this, researchers can adjust prior parameters (e.g., the strength of the Poisson prior) during the BRS process to limit the final number of rules, or post-process the results by filtering based on the importance of the rules (coverage, prevalence), striking a balance between feasibility and interpretability. To further reduce redundant rules generated by BRS, especially when the number of rules becomes excessive, LASSO regression can help further filter the most important rules for the outcome and avoid overfitting.

Finally, for variables that still need to be independently controlled in the regression stage, researchers should be mindful of their potential collinearity with the “BRS rule columns.” If some control variables have already been included in the BRS rule conditions, the correlation between these control variables and the rule indicators in the observations may be quite high, increasing the risk of multicollinearity in the regression. One feasible approach is: if a particular control variable appears repeatedly in the rules and conflicts with its own presence in the regression, the researcher can consider excluding this control column from the regression stage or merging the rules to avoid excessive collinearity that could harm the robustness of the model.

## 5. Discussion of Results and Implications

This study, grounded in the work, nonwork, and sleep (WNS) framework and conservation of resources (COR) theory, combined with the Bayesian rule set (BRS) algorithm, investigates the complex interplay between sleep, job satisfaction, and various other factors. The results indicate that higher job satisfaction is associated with two distinct patterns: (1) older individuals with medium to high sleep quality and shorter working hours, and (2) individuals with medium to high health levels, high autonomy, and shorter working hours. These patterns suggest that sleep, influenced by work-related factors such as hours worked and autonomy, plays a key role in shaping job satisfaction.

However, it is crucial to recognize that these findings may be significantly shaped by the broader context of work culture and professional background, especially within the Chinese context. The Chinese work environment, characterized by cultural elements such as collectivism, authoritative leadership, and high performance expectations, creates a distinctive backdrop for understanding how sleep interacts with job satisfaction (Kwan et al., 2024). For instance, in urban areas of China, employees—particularly those working in high-intensity industries—may face substantial workloads due to extended working hours and heightened job pressure. These factors not only disrupt sleep but also lead to a direct decline in job satisfaction (Chen et al., 2023; Zheng et al., 2023). In addition, other factors such as social class, income inequality, and urban–rural disparities may further complicate this relationship by influencing access to resources, work conditions, and overall well-being (Li, 2017; McGuffog et al., 2023). Therefore, a nuanced understanding of the interaction between these factors is essential to fully comprehend how they collectively shape sleep and job satisfaction in different societal and cultural contexts.

Furthermore, it is important to consider how occupational types—such as office work and technical labor—exhibit distinct patterns in terms of sleep and job satisfaction. Office work, characterized by higher cognitive demands, often involves irregular work schedules, including unexpected overtime, unclear end-of-work times, and unpredictable workloads. These job characteristics, coupled with the high mental effort required, significantly contribute to sleep disturbances, making it difficult for workers to disconnect from work and recover during nonwork hours. Existing studies support this notion, showing that cognitively demanding jobs tend to increase psychological demands, resulting in prolonged cognitive arousal that negatively impacts sleep (Caldwell et al., 2019). In contrast, physical labor, which typically involves more structured work hours and tasks, provides clearer boundaries between work and personal life, which may reduce cognitive load and improve the ability to separate work from personal time. However, the physical demands of such jobs can result in fatigue that also impacts sleep over time.

Moreover, recent research into work hours and productivity suggests that reducing work hours while maintaining the same pay can help maintain or even improve productivity. For example, Beauregard & Henry (2009) demonstrated that shorter work hours can enhance work–life balance and increase job satisfaction without compromising productivity. This finding aligns with studies on workweek reduction, which suggest that fewer workdays can lead to improved well-being and reduced burnout, ultimately benefiting both employees and organizations (Jahal et al., 2024). Thus, organizations should consider adopting flexible work arrangements that prioritize shorter work hours with full compensation, as this could foster a more engaged, satisfied, and productive workforce.

Lastly, the transformative impact of modern workplace communication technologies cannot be overlooked. The rise of workplace connectivity has led to an “always available” phenomenon, blurring the boundaries between work and personal life and contributing to increased stress and disrupted sleep patterns. Research by Seedoyal Doargajudhur and Hosanoo (2023) highlights that the constant connectivity associated with modern technologies can lead to prolonged cognitive activation beyond regular working hours. This in turn disrupts circadian rhythms, increases screen exposure, and elevates workplace anxiety, all of which significantly affect sleep. These challenges are particularly pronounced in knowledge-intensive sectors, where cognitive demands are high. In contrast, for employees in technical fields, especially those in physically demanding jobs, although their working hours may be more fixed, the physical strain of the work can result in fatigue that negatively impacts sleep. As such, different professional backgrounds and work cultures likely have varying influences on employee sleep and job satisfaction, suggesting a need for future research that further explores these relationships.

### 5.1. Theoretical Contributions

First, while the WNS framework emphasizes the critical role of sleep quality in managing work and nonwork roles, especially in terms of resource and time management, its analysis has mainly focused on the overall relationships between these domains, offering little detailed examination of specific resource mechanisms (Crain et al., 2018). Additionally, although the WNS framework recognizes that attitudes, behaviors, and states in work and nonwork contexts affect sleep quality, it lacks data-driven validation. This study, through the use of the Bayesian rule set algorithm, deeply explored variable combinations centered on sleep quality, revealing how these combinations interact within specific resource mechanisms to effectively predict job satisfaction. By validating with empirical data, this study not only fills the gap in the WNS framework’s analysis of resource mechanisms but also provides a more profound understanding of the role of sleep quality in work and nonwork environments.

Second, although the conservation of resources (COR) theory explains the relationship between sleep and job satisfaction, its applicability in different contexts requires further validation, and the dynamic and complex nature of sleep as a vital resource has not been fully explored (Hobfoll, 1989, 2011; Hobfoll et al., 2018). To address this issue, this study used the Bayesian rule set algorithm to explore variable combinations centered on sleep quality that effectively predict high job satisfaction. The study further examined how sleep quality, as a resource, influences job satisfaction, thereby validating the applicability of COR theory in explaining the sleep–job satisfaction relationship.

Third, to address the ambiguity in the existing research regarding the causal relationship between sleep quality and job satisfaction, this study, using the Bayesian rule set algorithm, uncovered variable combinations centered on sleep quality (Litwiller et al., 2017; Qiu et al., 2022). This method identifies complex interaction relationships between variables rather than just a single causal path. By analyzing multiple variable combinations, this study more clearly defines the rule by which sleep quality, as a predictor variable, affects job satisfaction. This provides a reference for future research in accurately revealing the causal relationship between the two.

Fourth, the heterogeneity of samples (such as differences in gender and health status) often leads to inconsistent conclusions in existing research (Benjamins et al., 2017). Using the Bayesian rule set algorithm, this study systematically analyzed the interaction combinations among different groups, exploring the relationship between sleep quality and job satisfaction under varying conditions of health status, age, and autonomy. By introducing multidimensional variable combination analysis, this study effectively controlled for sample heterogeneity, ensuring the universality and robustness of the results.

Fifth, previous studies have faced inconsistencies and differences in mechanism effects due to the omission of important variables (Opoku et al., 2023; Henderson and Horan, 2021; Litwiller et al., 2017). In response, this study incorporated key control variables such as demographic variables and work–family conflict into the model (Kossek & Lautsch, 2018). This approach highlights the essential factors to consider when studying the relationship between sleep quality and job satisfaction. By including these previously overlooked variables, the study enhances the completeness and explanatory power of the model, addressing shortcomings in previous research related to explaining the sleep–job satisfaction relationship.

### 5.2. Practical Implications

First, older employees tend to have higher job satisfaction when they maintain good sleep quality and work fewer hours. This finding aligns with the studies by Artazcoz et al. (2009) and Bae (2021), which suggest that reducing working hours can help employees achieve a better balance between work and life, thereby improving overall well-being. It is important to clarify that reducing working hours should ideally be coupled with the same level of pay, as this would allow older employees to benefit from improved work–life balance without sacrificing income. In this context, organizations should provide flexible working arrangements, such as phased retirement plans or reduced working hours options, especially in the later stages of an employee’s career. This approach not only improves job satisfaction but also enhances employees’ overall health and work performance. If reducing hours leads to a reduction in pay, it may adversely affect job satisfaction, as employees could feel the trade-off between work–life balance and income stability is too great.

Second, employees with better health, high autonomy, and shorter working hours also exhibit higher job satisfaction. This finding is supported by the studies of Wheatley (2017) and Knudsen et al. (2007), which indicate that greater autonomy reduces work stress and improves both job satisfaction and sleep quality. Therefore, organizations should focus on increasing employees’ autonomy, particularly for those in good health. This can be achieved by giving employees more control over task allocation and work methods, while also providing a supportive work environment and access to health resources. Additionally, reducing their working hours—without reducing pay—can further boost satisfaction and productivity, as it enables better time management and recovery of energy.

Lastly, improving job satisfaction depends not just on a single factor but on the combination of multiple factors. Kossek and Lautsch (2018) pointed out that individuals with more resources (such as high autonomy and good health) are better able to manage the dual pressures of work stress and family responsibilities, maintaining high job satisfaction. Therefore, organizations should adopt integrated strategies to optimize the combination of multiple factors. For instance, by focusing on sleep quality, autonomy, and management of work hours (with unchanged pay), organizations can create a more supportive work environment, which will help to enhance overall employee satisfaction and organizational effectiveness.

### 5.3. Limitations and Future Research

First, this study primarily relied on self-reported data, which is susceptible to subjective biases. For instance, respondents may underreport their income due to privacy concerns or societal expectations, especially when it comes to sensitive topics such as financial information. Similarly, when reporting on job satisfaction, participants may be influenced by social desirability bias, providing answers they perceive as more socially acceptable rather than their true feelings. These biases could impact the accuracy of the data and limit the generalizability of the findings. To enhance the credibility of future research, it is recommended to incorporate objective data sources, such as company-provided salary records or performance evaluations, to complement self-reported measures (Akankunda et al., 2024). Additionally, physiological data, such as sleep quality monitored through wearable devices, could offer more objective insights into the relationship between sleep and job satisfaction (Perez-Pozuelo et al., 2020).

Second, while this study focused on sleep quality as the central variable, it primarily explored how other variables interact with sleep quality to influence job satisfaction. Future research could explore the role of other key variables, such as work–family conflict, as core factors affecting job satisfaction. Work–family conflict, for example, may vary across cultures and contexts, potentially influencing the work–sleep relationship in different ways (Zhang et al., 2023). Investigating how these factors interact with sleep quality could offer more comprehensive insights into the dynamics of job satisfaction.

Lastly, future research could benefit from adopting longitudinal research designs to track changes over time within the same group of employees. While existing research has provided valuable insights into the sleep–job satisfaction relationship (Lin et al., 2018; Sheng et al., 2018), there remains limited longitudinal evidence to establish clear directional relationships between these variables. The few available studies, such as Litwiller et al. (2017) and Qiu et al. (2022), suggest bidirectional influences, but more systematic longitudinal research is needed. This approach would provide a deeper understanding of the evolving relationship between sleep quality, job satisfaction, and other influencing variables over time (Ployhart & Vandenberg, 2010). Longitudinal studies could capture dynamic changes and help establish causal relationships, offering more robust insights into how these variables interact in real-world settings. For instance, examining how resource conservation or depletion (as framed in COR theory) influences sleep quality across different stages of employment could provide useful insights into causality.

## Figures and Tables

**Table 1:** Variable names and corresponding survey items.

Variable Name	English Abbreviation	Corresponding Survey Item	Variable Description
Job Satisfaction	Jobs	Overall, are you satisfied with your current job?	1 = Very dissatisfied; 2 = Dissatisfied; 3 = Neutral; 4 = Satisfied; 5 = Very satisfied (Reverse Coding of Items)
Work Time (Average)	workt	In the past month, how many hours did you work in the week with the shortest working hours? In the past month, how many hours did you work in the week with the longest working hours?	Both are completed by the respondent (specified to the minute)
Autonomy	Selfd	To what extent can you decide the specifics of your work in your current job?	1 = Fully autonomous; 2 = Partially autonomous; 3 = Slightly autonomous; 4 = Not autonomous at all (Reverse Coding of Items)
Work–Family Conflict	Workf	Your work interferes with your family life.	1 = Always; 2 = Often; 3 = Sometimes; 4 = Rarely; 5 = Never (Reverse Coding of Items)
Family–Work Conflict	Famw	Your family life interferes with your work.	1 = Always; 2 = Often; 3 = Sometimes; 4 = Rarely; 5 = Never (Reverse Coding of Items)
Commute Time	Roadt	How much time (in minutes) does it take you to commute from your home (or where you live) to your workplace (one way)?	Completed by the respondent (specified to the minute)
Income	income	What was your total personal occupational income for the year 2020 (in RMB)?	Completed by the respondent
Social Class	class	In our society, some people are at the top and some are at the bottom. This ladder represents these levels. ‘10’ is the top and ‘1’ is the bottom. Where do you place yourself on this ladder?	Scored by the respondent on a 10-level scale, representing scores from 1 to 10, with higher scores indicating a higher social class
Fairness	fair	Overall, do you think today’s society is fair?	1 = Completely unfair; 2 = Somewhat unfair; 3 = Neither fair nor unfair; 4 = Somewhat fair; 5 = Completely fair
Trust in Others	Trusto	Overall, do you agree that most people in this society can be trusted?	1 = Strongly disagree; 2 = Somewhat disagree; 3 = Neither agree nor disagree; 4 = Somewhat agree; 5 = Strongly agree
Sleep Quality	Sleepq	In the past month, how would you rate the quality of your sleep?	1 = Very good; 2 = Good; 3 = Poor; 4 = Very poor (Reverse Coding of Items)
Sleep Time	sleept	How long do you usually sleep on weekdays (this does not refer to the total time in bed, and does not include naps)? (hours + minutes)	Completed by the respondent (specified to the minute)
Marital Status	marry	What is your current marital status?	1 = Single; 2 = Cohabiting; 3 = First marriage with spouse; 4 = Remarried with spouse; 5 = Separated but not divorced; 6 = Divorced; 7 = Widowed
Gender	gender	Gender (recorded by the interviewer)	1 = Male; 2 = Female
Part-Time Jobs	Partj	Do you currently hold more than one job?	1 = No; 2 = Yes
Age	age	What is your birthday?	Completed by the respondent (specified to year, month, and day)
Education Level	edul	What is your highest level of education?	1 = No formal education; 2 = Traditional private school/Literacy class; 3 = Primary school; 4 = Junior high school; 5 = Vocational high school; 6 = General high school; 7 = Secondary specialized school; 8 = Technical school; 9 = Associate degree (Adult higher education); 10 = Associate degree (Regular higher education); 11 = Bachelor’s degree (Adult higher education); 12 = Bachelor’s degree (Regular higher education); 13 = Postgraduate and above; 14 = Other (please specify: _________)
Health Status	health	How would you rate your current health status?	1 = Very unhealthy; 2 = Somewhat unhealthy; 3 = Average; 4 = Somewhat healthy; 5 = Very healthy

Note: Abbreviations were used to facilitate R analysis and correspond to the output variables in R.

**Table 2:** Descriptive statistics of variables.

Variable	Obs.	Mean	Std. Dev.	Min.	Max.
Income	618	78,750.76	129,000	0	1,500,000
Health Status	618	3.841	0.862	1	5
Trust in Others	618	3.649	0.924	1	5
Fairness	618	3.426	0.892	1	5
Social Class	618	4.502	1.648	1	10
Autonomy	618	2.366	0.848	1	4
Commute Time	618	27.53	40.441	0	480
Work–Family Conflict	618	4.089	1.031	1	5
Family–Work Conflict	618	4.427	0.737	1	5
Job Satisfaction	618	2.304	0.816	1	5
Sleep Quality	618	2.034	0.629	1	4
Work Time	618	48.35	17.056	0	84
Sleep Time	618	7.441	0.935	6	12
Age	618	40.066	11.443	18	65
Marital Status	618	0.77	0.421	0	1
Gender	618	0.502	0.5	0	1
Education Level	618	3.11	1.479	1	5
Part-Time Jobs	618	0.071	0.257	0	1

## Data Availability

The data for this study originate from the Chinese General Social Survey (CGSS). The CGSS provides comprehensive user guidelines and resources. Access to the CGSS data requires an application, as direct sharing or redistribution by individuals is not permitted. Detailed application information and instructions are available upon account registration at http://www.cnsda.org.
